# Occupational Toluene Exposure Induces Cytochrome P450 2E1 mRNA Expression in Peripheral Lymphocytes

**DOI:** 10.1289/ehp.8192

**Published:** 2005-10-13

**Authors:** Ania Mendoza-Cantú, Fabiola Castorena-Torres, Mario Bermúdez de León, Bulmaro Cisneros, Lizbeth López-Carrillo, Aurora E. Rojas-García, Alberto Aguilar-Salinas, Maurizio Manno, Arnulfo Albores

**Affiliations:** 1 Sección de Toxicología and; 2 Departamento de Genética y Biología Molecular, Centro de Investigación y de Estudios Avanzados, National Polytechnical Institute, Mexico City, Mexico; 3 Centro de Investigación en Salud Poblacional, Instituto Nacional de Salud Pública, Cuernavaca, Morelos, Mexico; 4 Coordinación Nacional de Salud en el Trabajo, Centro Médico Nacional Siglo XXI, Instituto Mexicano del Seguro Social, Mexico City, Mexico; 5 Dipartimento di Scienze Mediche Preventive, Sezione di Tossicologia Occupazionale, Università degli Studi di Napoli “Federico II,” Naples, Italy

**Keywords:** chlorzoxazone, CYP2E1 mRNA induction, lymphocytes, occupational exposure, toluene

## Abstract

Print workers are exposed to organic solvents, of which the systemic toxicant toluene is a main component. Toluene induces expression of cytochrome P450 2E1 (CYP2E1), an enzyme involved in its own metabolism and that of other protoxicants, including some procarcinogens. Therefore, we investigated the association between toluene exposure and the CYP2E1 response, as assessed by mRNA content in peripheral lymphocytes or the 6-hydroxychlorzoxazone (6OH-CHZ)/chlorzoxazone (CHZ) quotient (known as CHZ metabolic ratio) in plasma, and the role of genotype (5′-flanking region *Rsa*I/*Pst*I polymorphic sites) in 97 male print workers. The geometric mean (GM) of toluene concentration in the air was 52.80 ppm (10–760 ppm); 54% of the study participants were exposed to toluene concentrations that exceeded the maximum permissible exposure level (MPEL). The GM of urinary hippuric acid at the end of a work shift (0.041 g/g creatinine) was elevated relative to that before the shift (0.027 g/g creatinine; *p* < 0.05). The GM of the CHZ metabolic ratio was 0.33 (0–9.3), with 40% of the subjects having ratios below the GM. However, the average CYP2E1 mRNA level in peripheral lymphocytes was 1.07 (0.30–3.08), and CYP2E1 mRNA levels within subjects correlated with the toluene exposure ratio (environmental toluene concentration:urinary hippuric acid concentration) (*p* = 0.014). Genotype did not alter the association between the toluene exposure ratio and mRNA content. In summary, with further validation, CYP2E1 mRNA content in peripheral lymphocytes could be a sensitive and noninvasive biomarker for the continuous monitoring of toluene effects in exposed persons.

Solvents are complex chemical mixtures containing many different hydrocarbon types, such as alkanes, alcohols, ketones, aldehydes, esters, ethers, and small aromatic molecules, that evaporate and become incorporated into environmental air as volatile organic compounds (VOCs). Although the percentage of each chemical in solvent formulations varies among commercial brands, toluene is ubiquitously the most abundant ingredient (Mercado-Calderón 1997). Products used in many industrial workshops and in the home contain potentially hazardous solvents. Therefore, people working in such industries or at home risk continuous exposure to VOCs released from solvents.

Toluene is a systemic toxicant that affects liver, kidney, and the central nervous system [Agency for Toxic Substances and Disease Registry (ATSDR) 2000], the last being considered the main target organ (Byrne et al. 1991). Toluene biotransformation takes place in the liver by the cytochrome P450 2E1 (CYP2E1) isoform, producing hippuric acid, which is excreted in urine (Nakajima and Wang 1994). Toluene also up-regulates its own metabolism by inducing CYP2E1 activity in rat liver and peripheral lymphocytes (Gonzalez-Jasso et al. 2003). CYP2E1 induction is relevant because this enzyme activates many xenobiotics to become ultimate toxicants (Guengerich and Shimada 1991). In fact, CYP2E1 induction is the first step leading to the development of certain chemically mediated cancers (Lieber 1997). Consequently, exposure to toluene in an occupational setting could represent an increased risk of toxicity. *CYP2E1* polymorphisms may also modulate this condition, although the extent to which 5′-flanking region *Rsa*I/*Pst*I polymorphisms alter induction is not clear (Wormhoudt et al. 1999).

Because of the significant health risks associated with high levels of solvent exposure, routine monitoring of exposed subjects and their work environment is necessary to ensure good occupational health and safety conditions. It is thus advisable to perform studies at multiple levels to elucidate the mechanisms involved in mediating occupational chemical exposure effects, and to search for earlier and more sensitive biomarkers that will improve occupational safety (Mutti 1999). Accordingly, several studies have been reported with the aim of assessing environmental and health conditions of VOC-exposed workers (Apostoli et al. 1982; Wallen 1986); CYP2E1 phenotype determination in such populations may provide additional information.

Chlorzoxazone (CHZ) is a centrally acting muscle relaxant drug that has been used extensively as a selective probe for CYP2E1 phenotyping; this isoform is the main enzyme involved in its 6-hydroxylation (6OH-CHZ) in humans (Lucas et al. 1999). However, because CHZ clinical prescription is no longer available in Europe and is under revision in the United States (Lucas et al. 2001), it should no longer be used as a routine monitoring probe (Tanaka et al. 2003). Therefore, alternative methods to assay CYP2E1 phenotype *in vivo* are being investigated. Raucy et al. (1997) reported a relationship between CYP2E1 activity in peripheral blood lymphocyte microsomes and plasma CHZ metabolic ratio (6OH-CHZ/CHZ quotient; CYP2E1 metabolism indicator) in alcoholic subjects. This association suggests that peripheral lymphocytes and liver are regulated similarly and could be advantageous for monitoring purposes (Gonzalez-Jasso et al. 2003). Little information, however, is available about the effects of environmental VOCs on CYP2E1 regulation in human peripheral lymphocytes. Consequently, we investigated the association of toluene exposure with both CYP2E1 mRNA content in peripheral lymphocytes and CHZ 6-hydroxylation in occupationally exposed subjects. We further assessed the contribution of the 5′-flanking region *Rsa*I/*Pst*I polymorphism in measured lymphocyte CYP2E1 mRNA content and CHZ 6-hydroxylation.

## Materials and Methods

### Study design.

A cross-sectional study was performed with print industry workers in Mexico City. The study population consisted of 103 unrelated male print workers employed as flat or rotary printing press operators (24%), operator assistants (33%), ink handlers (22%), machine maintenance personnel (mechanics and electricians) (13%), or supervisors (8%). Workers were exposed to a commercial non-benzene-containing organic solvent, composed of a complex mixture of VOCs, of which toluene and xylene were the main constituents. Inclusion criteria for participants were 2-fold: *a*) only clinically healthy males with normal hepatic function test values were included, and *b*) only those not taking drugs that alter CYP2E1 activity (e.g., acetaminophen, isoniazid, disulfiram, chlormethiazole, or chloroquine) were included (Frye and Branch 2002; Park et al. 1993; Simi and Ingelman-Sundberg 1999).

Workers were contacted through the National Coordination of Health at the Workplace (Instituto Mexicano del Seguro Social) and received a thorough explanation of the aims and procedures of this investigation; participants signed an informed consent form (response ratio, 85%). Subjects were directly interviewed by trained personnel in order to record clinical, occupational, and sociodemographic characteristics, tobacco and alcohol consumption, and use of personal protection equipment. In addition, the body mass index (BMI) was calculated for each subject as the quotient of body weight divided by the square of the height (Brown 1995). Subjects were instructed to avoid smoking and alcohol consumption for 5 days before the sample collection (Plee-Gautier et al. 2001). The CINVESTAV-IPN Ethical Committee approved this investigation protocol.

### Quantitation of toluene levels in the environment.

Personal monitors measured toluene environmental levels in the indoor air of working areas. Each worker wore a Radiello passive diffusion monitor (code 130; Fondazione Salvatore Maugeri, Padua, Italy) placed within the respiratory area, for an 8-hr work shift. To extract absorbed solvent molecules, 2 mL carbon disulfide was applied to the monitors, and they were placed on the laboratory bench for 30 min and given an occasional shake. Extracts were analyzed by gas chromatography/mass spectrometry, using a Finnigan chromatograph (San Jose, CA, USA) equipped with a PONA capillary column (50 m × 0.25 mm inner diameter × 0.25 μm film thickness; stationary phase, 5% phenyl-methyl silicone) and a flame ionization detector (FID). Run conditions were as follows: initial temperature was 40°C; then the temperature was increased at an initial ramp of 10°C/min to 150°C and a second ramp of 15°C/min to 180°C (2 min). The injector (splitless mode) temperature was 200°C, the FID temperature was 250°C, and the carrier gas (helium 99.998%; Praxair, Mexico City, Mexico) was sustained at a constant flow rate of 35 mL/min. We identified and quantified the chemicals present in the extract samples by comparison with standards (ChemService, West Chester, PA, USA) (Cocheo 1983).

### Biologic sampling.

Blood samples were collected from each subject’s radial vein and placed in tubes with or without EDTA for lymphocyte isolation and CHZ metabolism evaluation or liver function tests (serum), respectively. Urine samples were obtained at the beginning and end of an 8-hr work shift and used to measure hippuric acid levels. Liver function was assessed in serum by measuring alanine aminotransferase, aspartate aminotransferase, and gamma-glutamyl transferase activities (Bergmeyer and Horder 1980). Creatinine was measured in urine by the Jaffe reaction method (Bartels and Bohmer 1972).

### Quantitation of urinary hippuric acid.

We measured urinary hippuric acid according to the recommendations of the National Institute for Occupational Safety and Health (NIOSH 1994). Briefly, we acidified a 1-mL urine sample by adding 40 μL hydrochloric acid, saturated the solution with 0.3 g sodium chloride, and then extracted hippuric acid by combining the solution with 4 mL ethyl acetate and shaking the mixture for 5 min. After centrifugation at 3,000 rpm for 10 min, the organic layer was evaporated under a nitrogen stream until dry and then redissolved in 1 mL distilled water. Recovery percentage was calculated by addition of a hippuric acid standard to urine samples; this control calculation with the standard revealed a 95% mean recovery rate.

Urine extracts were analyzed by high-performance liquid chromatography (HPLC) with a Perkin Elmer 235C chromatograph (PerkinElmer, Norwalk, CT) equipped with a diode array detector and a 50 μL loop. Samples were separated in a 250 mm × 5 mm Spherisorb Ps phaseb column packed with 5 μm C_18_ reversed phase (Phase Separation Inc., Norwalk, CT, USA). The mobile phase was a mixture of acetonitrile and 0.2% glacial acetic acid in water (10:90 vol/vol) and was maintained at a flow of 1 mL/min. The wavelength setting was 254 nm.

### Quantitation of CHZ and 6OH-CHZ in plasma.

After a 12-hr fast, volunteers ingested a CHZ tablet (500 mg; Barr Laboratories, Pomona, NY, USA) and gave blood samples 2 hr later. Plasma was separated by centrifugation at 1,500 rpm for 10 min. CHZ and 6OH-CHZ were measured according to the methods described by Lucas et al. (1993). Plasma samples (0.5 mL) were digested overnight at 37°C with 20 μL *Helix pomatia* juice (132,000 units β-glucuronidase/mL; Sigma Chemical Co., Mexico City, Mexico) to hydrolyze 6OH-CHZ conjugates. Proteins were subsequently precipitated by addition of 0.6 N perchloric acid (4 mL). After centrifugation at 3,000 rpm for 10 min, CHZ and 6OH-CHZ were extracted from the supernatant twice with ethyl acetate (4 mL) by shaking for 90 min and centrifugation at 3,000 rpm at 4°C for 10 min. The organic layer was isolated, allowed to evaporate under a nitrogen stream until dry, and then resuspended in 0.5 mL of mobile phase. Recovery percentage was calculated in bovine serum samples with added CHZ and 6OH-CHZ standards that were analyzed simultaneously with the study samples.

CHZ and 6OH-CHZ levels were measured by HPLC using a Lambda-Max model 481 chromatograph (Waters Co., Milford, MA, USA) equipped with an ultraviolet detector and a 20 μL loop, and compound separation was carried out using a 250 mm × 5 mm Spherisorb Ps phaseb column, packed with 5 μm C_18_ reversed phase (Phase Separation Inc.). The mobile phase contained acetonitrile, 0.5% glacial acetic acid, and water (30:70 vol/vol) and was maintained at a flow rate of 1 mL/min. The wavelength setting was 287 nm. This procedure achieved a 65% mean recovery rate for CHZ and 6OH-CHZ, and the CHZ metabolic ratios reported were calculated.

### Evaluation of peripheral lymphocyte CYP2E1 mRNA content.

#### Isolation of total RNA.

Lymphocytes were separated from 7 mL of whole blood as described by Boyum (1968). Briefly, blood diluted 1:1 with saline solution was layered over 5 mL Lymphoprep (Nycomed Pharma AS, Oslo, Norway). After centrifugation at 1,600 rpm for 25 min at 25°C, we transferred the cell interface to a new tube, washed it twice with saline solution, and then centrifuged it at 1,200 rpm for 10 min at room temperature. Finally, the supernatant was discharged, and cells were prepared for RNA extraction treatment. Total RNA was extracted using TRIzol reagent (Gibco BRL, Life Technologies, Rockville, MD, USA) following the manufacturer’s instructions. Extracted RNA samples were suspended in diethylpyrocarbonate-treated water (20 μL). Total RNA was spectrophotometrically quantified, and its integrity was visualized by electrophoresis on 1.5% agarose gels stained with ethidium bromide.

#### Preparation of standard RNA.

**Figure 1:**
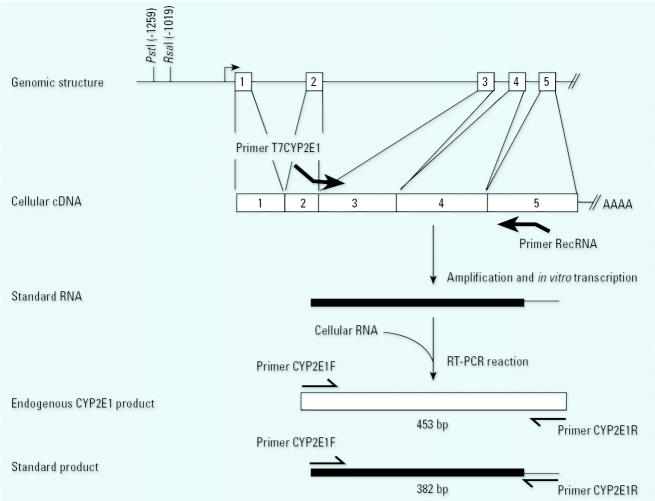
Schematic representation of the *CYP2E1* gene showing the polymorphism location and the design of the recRNA. For the genomic structure, exons, indicated by boxes, are numbered, and introns are represented by horizontal lines. The arrow indicates the transcription start site, and the *Rsa*I/*Pst*I polymorphism sites are denoted. To amplify the region of the *CYP2E1* gene encompassing exons 2–5, we used forward primer T7CYP2E1 on the junction of exons 2–3 and reverse primer RecRNA in exon 5. The presence of the T7 promoter sequence at the 5′ end of the forward primer enabled the recRNA to be produced by *in vitro* transcription. Cellular RNA and recRNA were co-reverse transcribed and amplified simultaneously with the CYP2E1F and CYP2E1R primers. Because the 20 bp stretch incorporated at the 3′ end of the primer RecRNA (thin line on standard) is complementary to primer CYP2E1, the RT-PCR reaction yielded products of 453 bp from the cellular RNA and of 382 bp from the recRNA. Both PCR products had the same DNA sequence, except for the 71 bp deletion in the middle of the standard fragment.

A set of primers was designed based on the human *CYP2E1* cDNA sequence (GenBank accession number NM000773; National Center for Biotechnology Information, Bethesda, MD, USA) to amplify a 443-bp fragment of the *CYP2E1* cDNA. The forward primer CYP2E1F (5′-ACAGGGACAGGGGAATCAT-3′) is located at the junction of exons 2 and 3, and the reverse primer CYP2E1R (5′-TGGGGTCCAGAGATTGATG-3′) is located within exon 5. Using modified versions of these primers, we constructed a standard RNA (recRNA) of the same length and sequence as endogenous CYP2E1 mRNA, except for a 71-bp deletion in the middle of the sequence. Construction of this competitor was accomplished in two steps. In the first step, primers T7CYP2E1 (5′-TAATACGACTCACTATAGGACAGGGACAGGGGAATCAT-3′) and recRNA (5′-TGGGGTCCAGAGATTGATGCAGGCAAGTAGTGTAGAAAG-3′) were used to amplify a cDNA from human peripheral blood lymphocytes. Primer T7CYP2E1 contains the T7 promoter sequence at the 5′-end of primer CYP2E1F (underlined sequence), and primer recRNA corresponds to CYP2E1R with the exception of a mismatching stretch of 20 bp located at the 3′-end (underlined sequence). In the human *CYP2E1* cDNA sequence, the 20-bp stretch maps 98 bp upstream from the CYP2E1R sequence, but in the recRNA primer both sequences were linked together to allow the further generation of the deleted polymerase chain reaction (PCR) fragment (Figure 1). The PCR product was purified with a column (Freeze ’N Squeeze DNA gel extraction spin column; BioRad Laboratories, Hercules, CA, USA). In the second step, recRNA derived from the PCR fragment containing the T7 promoter sequence was prepared by *in vitro* transcription with a T7 Transcription Kit (Roche Diagnostics GmbH, Mannheim, Germany), according to the manufacturer’s instructions. The recRNA was subsequently treated with RNase-free DNase (Boehringer Mannheim, Mannhein, Germany) to remove DNA template. recRNA was quantified by measuring its absorbance at 260 nm.

#### Quantitative reverse transcriptase (RT)-PCR.

**Figure 2:**
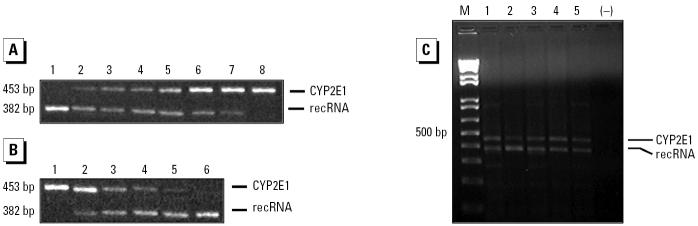
Reliability of the competitive RT-PCR assay for CYP2E1 transcript. (*A*) A fixed amount of human lymphocyte total RNA (5 μg) was incubated with decreasing amounts of recRNA (lane 1, 1 ng; lane 2, 100 pg; lane 3, 10 pg; lane 4, 1 pg; lane 5, 100 fg; lane 6, 10 fg; lane 7, 1 fg; lane 8, without recRNA. The expected PCR products of 453 and 382 bp correspond to endogenous CYP2E1 mRNA and recRNA, respectively. (*B*) A fixed amount of recRNA (1 pg) was incubated with decreasing amounts of human lymphocyte total RNA. Lane 1, 10 μg; lane 2, 7.5 μg; lane 3, 5 μg; lane 4, 2.5 μg; lane 5, 1 μg; lane 6, without RNA. (*C*) Representative gel of the competitive RT-PCR assay showing the CYP2E1 mRNA expression of different subjects (5 μg of total RNA and 10 pg of recRNA; lanes 1–5). Abbreviations: –, control with RNA sample omitted; M, DNA ladder.

Endogenous RNA and recRNA were co-reverse transcribed and amplified simultaneously. Reverse transcription reactions were performed in a final volume of 21 μL containing 16 mM Tris-HCl (pH 8.4), 40 mM KCl, 0.4 mM dithiothreitol, 4 mM MgCl_2_, 0.4 mM of each deoxynucleotide triphosphate, 0.5 μg oligo(dT), 40 U recombinant ribonuclease inhibitor RNaseOUT (Invitrogen, Carlsbad, CA, USA), and variable amounts of cellular RNA and recRNA. Sample solutions were incubated at 65°C for 10 min, 42°C for 50 min, and 70°C for 15 min. Fifty units of Superscript II reverse transcriptase was then added to each sample, and the samples were incubated at 42°C for 50 min and 70°C for 15 min. The reaction was terminated by rapid chilling on ice and by adding 4 U *Escherichia coli* RNase H (Invitrogen). The cDNA samples were amplified with primers CYP2E1F and CYP2E1R in 25 μL PCR buffer (100 ng of each primer, 1 U Taq polymerase, 200 mM Tris-HCl, pH 8.4, 500 mM KCl, 50 mM MgCl_2_, and 0.2 mM dNTP mixture), using the following PCR conditions: an initial denaturing step at 95°C for 5 min followed by 37 cycles of amplification that consisted of a denaturing step at 95°C for 30 sec, an annealing step at 56°C for 30 sec, and an extension step at 72°C for 30 sec. The reaction was terminated with a final extension step at 72°C for 5 min. All reactions were carried out in a Thermocycler GeneAmp PCR System 2400 (Applied Biosystems, Foster City, CA, USA). The RT-PCR reaction yielded a 453 bp fragment from endogenous RNA and a 382 bp fragment from recRNA (Figure 2). The CYP2E1 mRNA ratio was obtained by dividing the intensity of the endogenous mRNA band by the intensity of recRNA band.

### DNA sequencing.

To verify the authenticity of cellular and standard PCR products, they were sequenced in an ABI PRISM 310 Genetic Analyzer using the ABI PRISM Big Dye DNA sequencing kit (Applied Biosystems).

### CYP2E1 *genotype*.

*CYP2E1* genotype was assessed by restriction fragment length polymorphism analysis for the 5′-flanking region *Rsa*I/*Pst*I polymorphic sites (Figure 1), according to the method described by Hayashi et al. (1991).

### Statistical analysis.

Differences between initial and final hippuric acid concentrations were compared by Student’s *t*-test. Log transformations improved normality for CYP2E1 activity and urinary hippuric acid concentrations. Simple and multiple linear regression analyses were carried out to determine whether there were any associations between toluene exposure and CYP2E1 phenotype (enzymatic activity or mRNA content). These relationships were stratified by *Rsa*I/*Pst*I polymorphic site alleles. Multiple regression models were adjusted by age, BMI (classified as normal, overweight, and obesity), current drug consumption (categorized as yes or no), smoking habit (smokers or nonsmokers), and alcohol consumption (ranked as never, < 1 serving/week; seldom, 1 to 2 servings/week; frequent, > 2 servings/week). All statistical analyses were conducted using the STATA 8.0 software package (Stata Corporation, College Station, TX, USA).

## Results

### Demographic and health characteristics of the study population.

Among 103 male workers, 6 subjects were excluded because of abnormal liver function. The remaining 97 subjects had a mean age of 34.2 years (range, 18–63 years) and a mean employment time of 3.5 years (range, 1–9 years). The BMI data indicated that 70% of the subjects exhibited some degree of obesity. In addition, 55% of the study subjects were smokers, 92% of them declared some degree of alcohol consumption, and 10% considered themselves to be frequent drinkers. Table 1 summarizes the anthropometric characteristics and habits of the study population.

### Environmental toluene and urinary hippuric acid levels.

Table 2 summarizes the indoor air toluene concentrations to which the workers were exposed in the print workshop and the levels of toluene exposure biologic markers. Air toluene concentrations monitored individually for a work shift had a geometric mean (GM) of 52.80 ppm (range, 10–760 ppm). Thus, 54% of the subjects studied were exposed to toluene levels that exceeded the 50 ppm maximal permissible exposure level (MPEL) (NOM-010-STPS-1999NOM-010-STPS-2000).

**Figure 3:**
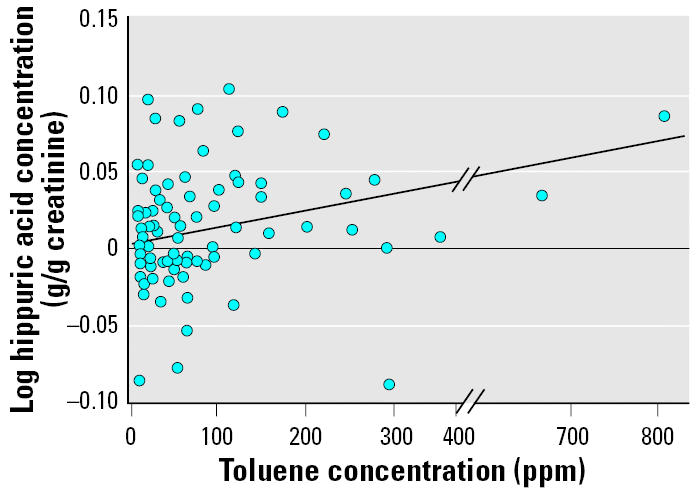
Effect of environmental toluene concentration on urinary hippuric acid concentration evaluated by simple linear regression analysis (*n* = 84). *y* = 0.0076*x* − 0.0133; *r*^2^ = 0.049; *p* = 0.041.

The GM of the urinary hippuric acid at the beginning and the end of a work shift were 0.027 and 0.041 g/g creatinine, respectively. Both values were below the maximal biologic permissible level (MBPL) of 2.5 g/g creatinine (NOM-047-SSA1-1993NOM-047-SSA1-1996). However, these data represent a 48% increase in urinary hippuric acid GM from the beginning to the end of the shift (*p* < 0.05) (Figure 3). This increase is consistent with the occurrence of active toluene absorption and biotransformation during the work shift.

### CY2E1 mRNA associations with toluene, hippuric acid, and the toluene exposure ratio.

**Figure 4:**
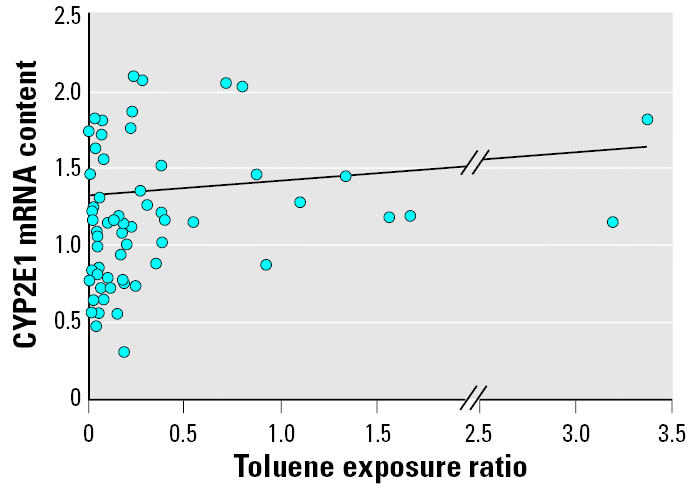
Relationship between toluene exposure ratio and CYP2E1 mRNA content (*n* = 67). *y* = 0.0929*x* + 1.320; *r*^2^ = 0.0854; *p* = 0.016.

In the present study, we assessed toluene exposure in two steps. First, toluene levels in work area air and hippuric acid levels in urine were measured for each subject, and these data were individually associated with the subject’s CYP2E1 mRNA data. These comparisons yielded statistically significant but opposite correlations. That is, environmental toluene correlated positively with CYP2E1 mRNA levels (*r*^2^ = 0.062, β= 0.011, *p* = 0.029; *n* = 76), whereas urinary hippuric acid levels correlated negatively with CYP2E1 mRNA levels (*r*^2^ = 0.065, β= −4.120, *p* = 0.027; *n* = 71). This dissociation may be due to the capacity of toluene to induce CYP2E1 mRNA expression, whereas hippuric acid reflects toluene disposition. Therefore, we conducted further analysis with the toluene exposure ratio, which was calculated as the quotient of each individual’s environmental toluene concentration exposure divided by his measured urine hippuric acid concentration. CYP2E1 mRNA level and the toluene exposure ratio showed a statistically significant relationship (*p* < 0.05) (Figure 4). Using this ratio enabled each subject’s exposure to be more accurately described, bearing in mind the variability between internal and external conditions among workers and allowing us to normalize the individual differences observed in toluene metabolism. Such inter-individual differences are likely to result from several factors: *a*) differing toluene exposures that lead to different hippuric acid production; *b*) a competitive inhibition of toluene metabolism by other VOCs, which was greater in workers exposed to high toluene concentrations; *c*) differing metabolic statuses, depending upon individual genetic characteristics and the extent of CYP2E1 induction; and *d*) possible development of a tolerance to toluene, as has been described for other CYP2E1 substrates (Shayiq et al. 1999). The GM for the toluene exposure ratio was 0.122, ranging between 0.007 and 3.366 (Table 2).

### CYP2E1 phenotype and genotype in the study population.

CHZ biotransformation is related to CYP2E1 activity (Lucas et al. 1999), which is induced by toluene (Uaki et al. 1995). However, CHZ metabolism can be competitively inhibited by other contaminants in an occupational setting (Lucas et al. 1999); such effects will be reflected in the CHZ metabolic ratio. The GM of the CHZ metabolic ratio was 0.33 (0–9.3); however, 40% of the study population values were < 0.3. On the other hand, blood peripheral lymphocyte CYP2E1 mRNA content was 1.07 with a range of 0.03–3.08 (Table 2).

Genotype frequencies for the *Rsa*I/*Pst*I polymorphisms were reported previously (Mendoza-Cantú et al. 2003). Briefly, 60 native homozygous (c1/c1), 34 heterozygous (c1/c2), and 3 homozygous (c2/c2) subjects were found. These genotype frequencies are in accordance with the Hardy-Weinberg equilibrium model (Klug and Cummings 2000).

The stratification of CYP2E1 phenotype parameters per genotype showed the following values: mRNA content c1/c1: GM = 1.0645; range = 2.069–0.304; c1/c2, GM = 1.056, range = 2.123–0.477; c2/c2, GM = 1.319, range = 1.708–1.153. The CHZ metabolic ratios for each genotype were as follows: c1/c1, GM = 0.327, range = 2.362–0.0023; c1/c2, GM = 0.367, range = 1.597–0.0310; c2/c2, GM = 0.430, range = 2.287–0.117. There was no apparent influence of genotype on the measured mRNA content levels and CHZ metabolic ratios.

### Relationships between toluene exposure and CYP2E1 phenotype.

We found a correlation between the toluene exposure ratio and CYP2E1 mRNA content (*r*^2^ = 0.0854, β= 0.0929, *p* = 0.016; *n* = 67) (Figure 4). However, the CHZ metabolic ratio did not correlate with either the toluene exposure ratio (*r*^2^ = 0.237, β= −0.007, *p* = 0.744; *n* = 84) or CYP2E1 mRNA content (*r*^2^ = 0.215, β = −0.026, *p* = 0.686; *n* = 78) (Table 3).

Obesity, age, and smoking, as well as consumption of alcohol and certain drugs, have been shown to modulate CYP2E1 expression (Bebia et al. 2004; Benowitz et al. 2003; Frye and Branch 2002; Lieber 2004; Park et al. 1993; Simi and Ingelman-Sundberg 1999). Additionally, the use of personal protective equipment can reduce exposure to toluene and other VOCs. To address their potential confounding effect on CYP2E1 expression by toluene occupational exposure, we performed a linear regression analysis (simple and multiple) by adjusting for the variables mentioned above. None of them had any appreciable effect on the relationship between the toluene exposure ratio and mRNA content (Table 4).

### Relation between toluene exposure ratio and CYP2E1 mRNA content stratified by genotype.

The relationship between toluene exposure ratio and CYP2E1 mRNA content was stratified according to *Rsa*I/*Pst*I polymorphisms, but this association was not modified by genotype in a slope analysis.

## Discussion

We investigated the effects of toluene exposure on expression of the CYP2E1 phenotype by measuring CYP2E1 mRNA induction in peripheral lymphocytes and CHZ metabolism in workers exposed to organic solvents and then determining whether these mRNA content and CHZ metabolic data were associated with genotype.

### Toluene in the work environment.

Toluene exposure in the present group of subjects (GM = 52.80 ppm) was to similar to the lower limit reported for toluene exposure in a previous study of workers from 25 Mexican companies (52.5–931 ppm) in which the workers routinely handled organic solvents, paints, and varnishes (Madrigal-Bujaidar et al. 1996). Regrettably, both studies revealed environmental toluene concentrations that exceeded the limits set by Mexican regulations (NOM-010-STPS-1999NOM-010-STPS-2000). Indeed in the present study, 54% of our study population was above the toluene MPEL. Therefore, stricter regulations should be implemented to ensure safe conditions in the occupational environment.

### Hippuric acid content in urine.

Hippuric acid has been used extensively as a toluene exposure biomarker, but this metabolite may occur in the diet, that is, via some fruits and food preservatives (Kawamoto et al. 1996). In this study, a statistically significant increase in hippuric acid concentrations during the work shift (48%) demonstrated active absorption and biotransformation of toluene by exposed workers. Despite the high toluene levels in air, the hippuric acid concentrations remained below the MBPL. This apparent inconsistency between the environmental and the biologic data leads to a disagreement between the MPEL and MBPL. Thus, an environmental exposure to toluene of 50 ppm for an 8 hr work shift resulted in a urinary hippuric acid concentration of < 2.5 g/g creatinine. De Bruin (1980) studied a group of volunteers exposed to 100 ppm toluene for 8 hr (twice the MPEL), but this high level of exposure produced a relatively low urinary hippuric acid excretion of 1.2 g/g creatinine. Although toluene is one of the main constituents of the organic solvent used in the print industry, these mixtures also contain many other VOCs that may be potential CYP2E1 substrates that may competitively inhibit its activity, as has been described for other chemicals (Campo et al. 1998; Tardif et al. 1993; Uaki et al. 1995).

### Toluene effects on CYP2E1 phenotype.

Various factors, including exposure to certain xenobiotics, can induce CYP2E1 expression and thereby result in an increase in its metabolic activity. Such induction could be a risk to the health of persons exposed to CYP2E1 protoxicant substrates; thus, CYP2E1 expression demands close monitoring in those individuals.

### The CHZ metabolic ratio.

Most of our subjects showed small CHZ metabolic ratios (40% had ratios < 0.3), indicating a very low CHZ biotransformation rate, a finding that may be attributable to competitive inhibition of CHZ metabolism by VOCs. We did not detect significant differences between the toluene exposure ratio and CHZ metabolic ratio, or between CHZ metabolism and mRNA content. Lucas et al. (1999) reported a decreased CHZ metabolic ratio in shoemakers exposed to glue and suggested that there may be metabolic competition between CHZ and the organic chemicals present in the solvent’s emissions. Furthermore, Ernstgård et al. (1999) found evidence that coexposure to acetone and CHZ or to toluene and CHZ increased plasma concentrations of both the organic chemicals and CHZ in human males, indicating that there was metabolic competition as a result of these interactions. In addition, Powell et al. (1998) found that human livers showed no correlation between mRNA levels and CYP2E1 protein content and *p*-nitrophenol and CHZ hydroxylation activity. Likewise, Sumida et al. (1999) reported no correlation between CYP2E1 mRNA level and CHZ hydroxylation in human liver biopsies. Thus, these lack of associations suggests that the CHZ metabolic ratio may not be an appropriate biomarker for toluene exposure when evaluated directly at the occupational site.

### CYP2E1 mRNA induction.

The associations of peripheral lymphocyte CYP2E1 mRNA content with environmental toluene or urinary hippuric acid were statistically significant. Furthermore, the peripheral lymphocyte CYP2E1 mRNA with the toluene metabolic ratio, a parameter that considers both exposure and disposition, was also significant.

The observed correlation between CYP2E1 mRNA induction and the toluene metabolic ratio suggests that CYP2E1 mRNA content is a much more sensitive probe than is the CHZ metabolic ratio. Moreover, CYP2E1 mRNA content is a safe, noninvasive test, for which samples can be obtained on site, and its expression in human peripheral lymphocytes appears to be regulated by the same factors as those affecting the liver (Raucy et al. 1997).

The CYP2E1 response we observed in this study demonstrated that toluene exposure affects CYP2E1 mRNA content, but more studies on toluene toxicity are needed to fully characterize the mechanism of induction. Other chemicals, such as ethanol, can regulate CYP2E1 activity by altering protein stabilization without affecting transcriptional activity (Roberts et al. 1995). Ethanol has also been shown to increase CYP2E1 gene expression at high doses (> 300 mg/dL blood) (Badger et al. 1993). Finally, *CYP2E1* genotype stratification did not modify the association between toluene exposure and mRNA content in peripheral lymphocytes.

In conclusion, our results suggest that CYP2E1 mRNA content may be a useful biomarker of physiologic effects in humans exposed to toluene. The mRNA response is the first step after its modulation and may not be affected by other factors that modify CYP2E1 enzyme activity, as has been suggested to be a complicating factor for CHZ metabolism. However, further studies are needed to fully characterize and validate the use of CYP2E1 mRNA content for health surveillance purposes.

## Correction

Values for the correlation between the toluene exposure ratio and CYP2E1 mRNA content were incorrect in the manuscript published online. They have been corrected here.

## Figures and Tables

**Table 1:** Anthropometric characteristics and habits of the study population.

Variable	Frequency or value
No.	97
Age (years)^a^	34.2 ± 9.8 (18–63)
Years employed^a^	3.5 ± 1.6 (1–9)
BMI (kg/m^2^)^a^	26.98 ± 4.15 (20.38–40.03)
Smoking status (%)^b^	
Nonsmoker	42 (43)
Smoker	55 (57)
Drinking status (%)	
Never	8 (8)
Seldom	79 (82)
Frequent	9 (10)

^a^Expressed as mean ± SD (range).

^b^Expressed as number of individuals (percentage of population).

**Table 2:** Environmental and biologic parameters in toluene-exposed subjects from the print industry.

Variable	No.	GM (range)
Environmental toluene (ppm)	94	52.80 (10–760)
Urinary hippuric acid (g/g creatinine)	90	
Beginning of the shift		0.027 (ND–0.167)
End of the shift		0.041* (ND–0.177)
Toluene exposure ratio^a^	84	0.122 (0.007–3.366)
CHZ metabolic ratio^b^	93	0.33 (0–9.3)
CYP2E1 mRNA content^c^	79	1.07 (0.30–3.08)

ND, not detected.

^a^Toluene exposure ratio (individual environmental toluene concentration/hippuric acid concentration quotient).

^b^CHZ metabolic ratio (6OH-CHZ/CHZ quotient).

^c^Expressed as average value.

* Statistically significant difference from concentrations at the beginning of the work shift (*p* < 0.05).

**Table 3:** Multiple regression analysis modeling the effects of toluene exposure on CYP2E1 phenotype and of CYP2E1 mRNA content on CHZ metabolic ratio.

	mRNA content				CHZ metabolic ratio			
Variable	β	*r*^2^	95% CI	n	β	*r*^2^	95% CI	n
Toluene exposure ratio	0.099	0.202	0.020 to 0.177	67	−0.007	0.237	−0.055 to 0.040	84
mRNA content					−0.026	0.215	−0.159 to 0.105	78

CI, confidence interval. Models were adjusted for alcohol and drug consumption, smoking habit, BMI, use of personal protection equipment, and age.

**Table 4:** Effects of potential confounders on CYP2E1 mRNA content.

Variable	β	*r*^2^	95% CI
Alcohol consumption	−0.034	0.0267	−0.081 to 0.012
Recent alcohol consumption	−0.025	0.0085	−0.086 to 0.036
Smoking habit	−0.125	0.0295	−0.289 to 0.037
Current drug consumption	−0.003	0.0002	−0.063 to 0.056
BMI	0.128	0.0451	−0.005 to 0.263
Use of protection equipment	0.024	0.0175	−0.017 to 0.065
Age	0.000	0.0003	−0.009 to 0.010

CI, confidence interval of multiple regression analysis.
